# An analysis of the services provided by community health workers within an urban district in South Africa: a key contribution towards universal access to care

**DOI:** 10.1186/s12960-021-00565-4

**Published:** 2021-02-18

**Authors:** L. S. Thomas, E. Buch, Y. Pillay

**Affiliations:** 1 Gauteng Department of Health, School of Health Systems and Public Health, University of Pretoria, and School of Public Health, University of Witwatersrand, Gauteng, South Africa; 2 School of Health Systems and Public Health, University of Pretoria and Colleges of Medicine, Gauteng, South Africa; 3 Formerly National Department of Health, Pretoria, South Africa

**Keywords:** Community health worker, Large-scale comprehensive care, Health and psychosocial activities

## Abstract

**Introduction:**

Community health worker teams are potential game-changers in ensuring access to care in vulnerable communities. *Who* are they? *What* do they actually do? Can they help South Africa realize universal health coverage? As the proactive arm of the health services, community health workers teams provide household and community education, early screening, tracing and referrals for a range of health and social services. There is little local or global evidence on the household services provided by such teams, beyond specific disease-oriented activities such as for HIV and TB. This paper seeks to address this gap.

**Methods:**

Descriptive secondary data analysis of community health worker team activities in the Ekurhuleni health district, South Africa covering approximately 280,000 households with 1 million people.

**Results:**

Study findings illustrated that community health workers in these teams provided early screening and referrals for pregnant women and children under five. They distributed condoms and chronic medication to homes. They screened and referred for hypertension, diabetes mellitus, HIV and TB. The teams also undertook defaulter and contact tracing, the majority of which was for HIV and TB clients. Psychosocial support provided was in the form of access to social grants, access to child and gender-based violence protection services, food parcels and other services.

**Conclusion:**

Community health workers form the core of these teams and perform several health and psychosocial services in households and poor communities in South Africa, in addition to general health education. The teams studied provided a range of activities across many health conditions (mother and child related, HIV and TB, non-communicable diseases), as well as social services. These teams provided comprehensive care in a large-scale urban setting and can improve access to care.

## Introduction

South Africa has to deliver on its Sustainable Development Goals, and as with other countries around the world, seeks to transform its health system to attain Universal Health Coverage [1]. Its National Health Insurance initiative is a means to achieve this [2]. One intervention to transform the health system is to re-organize and re-prioritize primary health care and district health services in the country, emphasizing disease prevention, control and health promotion. This is through a multi-pronged approach which includes community health worker (CHW) teams (known in South Africa as municipal ward based Primary Health Care Outreach teams/WBPHCOTs), Integrated School Health Services, District Clinical Specialist Teams; and contracting private providers such as General Practitioners. This is the core of the Primary Health Care re-engineering strategy for South Africa [3].

WBPHCOTs are fundamental ‘game changers’ in this regard [4]. These teams are the proactive arm of the health service, visiting households and communities to screen for diseases and risk factors and educate on basic health issues. WBPHCOTs, together with school health services, environmental health services, and health promotion play a critical role in strengthening community-based health services (Additional file 1: Appendix S1).

The 2020 Covid-19 pandemic highlighted the important role community health workers (CHWs) play in South Africa [5, 6], by improving Covid-19 health education and early screening. In the study district, over 1 million Covid-19 screening activities were conducted by CHWs over a 9-month period. CHWs are a means to provide equitable, affordable access to quality basic health services directly to the client [7]. Low and middle-income countries are increasingly strengthening their CHW programmes, viewing this as an affordable and critical intervention in attaining universal health coverage [8, 9].

## Background

In 2010–2011, South Africa (SA) launched its Primary Health Care (PHC) re-engineering strategy, of which the establishment of CHW teams, referred to as WBPHCOTs, was a critical component [10]. The strategy emanated from a ministerial technical task team’s visit to Brazil, where ‘Family health’ community-based teams had a significant impact on health outcomes of the sick and vulnerable [11, 12].

The CHW teams in South Africa were linked to specific clinics; the interface between health services and community [3]. Each clinic had a defined number of PHC outreach teams dependent on the population in a municipal ward. A municipal ward is the smallest political geographical demarcation in the 52 districts, country-wide. The local political ward representative would support these teams with community entry and engagement [13]. Each PHC outreach teams with ideally six CHWs would be responsible for 1500 to 2000 households (approximately 6000 population) in a ward; each CHW looking after approximately 250 households [14]. Each clinic supported four to five PHC outreach teams or more, depending on ward population. National guidelines stipulated that the CHWs should be recruited from the local community and be supervised and supported by a staff/enrolled nurse or a professional nurse serving as the Outreach Team Leader (OTL). The teams would be supported by other district resources such as health promoters, PHC trained clinicians, social workers and environmental health workers [15]. Depending on need of household members, a CHW would provide a range of comprehensive health and psychosocial services, emphasizing screening, disease prevention and health education for mothers and children (MCH), people with HIV/AIDS, Tuberculosis, chronic conditions such as hypertension or diabetes, and for orphan headed households. They would trace contacts and defaulters and refer patients back to the clinic to re-start treatment.

The Ekurhuleni health district where this study took place is one of three urban metropolitan municipalities in Gauteng province, a national economic hub [16] (Additional file 2: Appendix S2). Its population of approximately four million people live densely in a mix of affluent and vulnerable areas with formal and informal housing; 75% of the population are medically uninsured and dependent on the public health sector. It has a quadruple burden of disease, similar to the rest of South Africa, with high rates of maternal and infant mortality, HIV/AIDS, TB, chronic diseases of lifestyle, and violence related conditions [17].

CHWs are individuals who have a general understanding of their own communities’ language and culture, can provide culturally appropriate health services to the community and require shorter training than health professionals [18].

During apartheid in South Africa, there were limited numbers of CHW programmes and the roles of CHWs, while diverse, were restricted to few health conditions and programmes; these were not formally part of the health system, and may have been used as an excuse to not provide integrated and appropriate health care [19]. Due to a lack of support from the apartheid government, these cadres could not be sustained effectively [20]. Later, during the HIV epidemic in the early 1990s, newly emerging CHW programs funded through non-governmental organisations and government subsidies had a technical and disease oriented focus; which reduced the value these resources could have had on broader health and socio-economic outcomes. They made limited contributions to local health systems.

The national policy on CHWs has evolved and their roles have progressed since 2010–2011 with the introduction of CHW teams (WBPHCOTs) in providing a more comprehensive range of basic activities, across health conditions. Nationally, they have been variably implemented with approximately 70,000 CHWs utilized to improve health services [14]. However, there is no detailed analysis of what these CHWs do and their potential contribution to the health system, beyond their impact on HIV and TB programmes. While SA has routine primary health care service reporting, this is not done for CHW household services. This study describes these activities and contributes to local evidence in this regard.

**Fig. 1:**
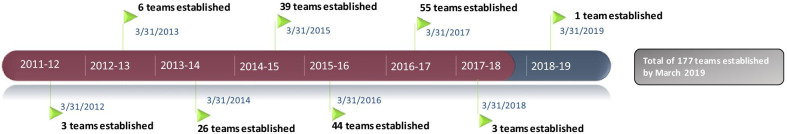
Time line of CHW teams established in Ekurhuleni

Starting in 2011–2012, the Ekurhuleni health district gradually established 177 teams by March 2019 (Fig. 1) at a cost of USD 5 million. The 177 teams consisted of 1108 CHWs and 155 OTLs providing 1 million people in 280,000 households with health education, early screening, tracing, referrals and linkage to care. The CHWs in the teams studied were each paid a stipend of USD 232/month, on fixed long-term contracts and had ongoing training based on need. By March 2019, the Ekurhuleni population coverage was just 25%. With additional funding and resources, more teams could have been established, but this was not available.

## Study objective and methods

The study objective was to document routine CHW outputs in Ekurhuleni households through secondary data analysis of team activities from 2016/17 to 2018/19.

Since 2016 CHWs have used locally developed paper-based tools to capture their daily activities. These are summarized daily, weekly and monthly by their OTLs and then collated into a monthly district report (Additional file 3: Appendix S3). This was further analysed into annual averages and proportions to discern trends.

## Results

Each CHW was allocated a designated number of households and provided services in these homes. Approximately 1.7 million headcount activities on screening, tracing, referrals and linkages to care services were provided in the 280,000 households over the 3-year study period. The annual averages per activity show that most of the services provided by CHWs were to identify risks and problems early. (Figs. 2, 3, 4, 5, 6, 7, 8, 9, 10, 11, 12 and 13).

**Fig. 2:**
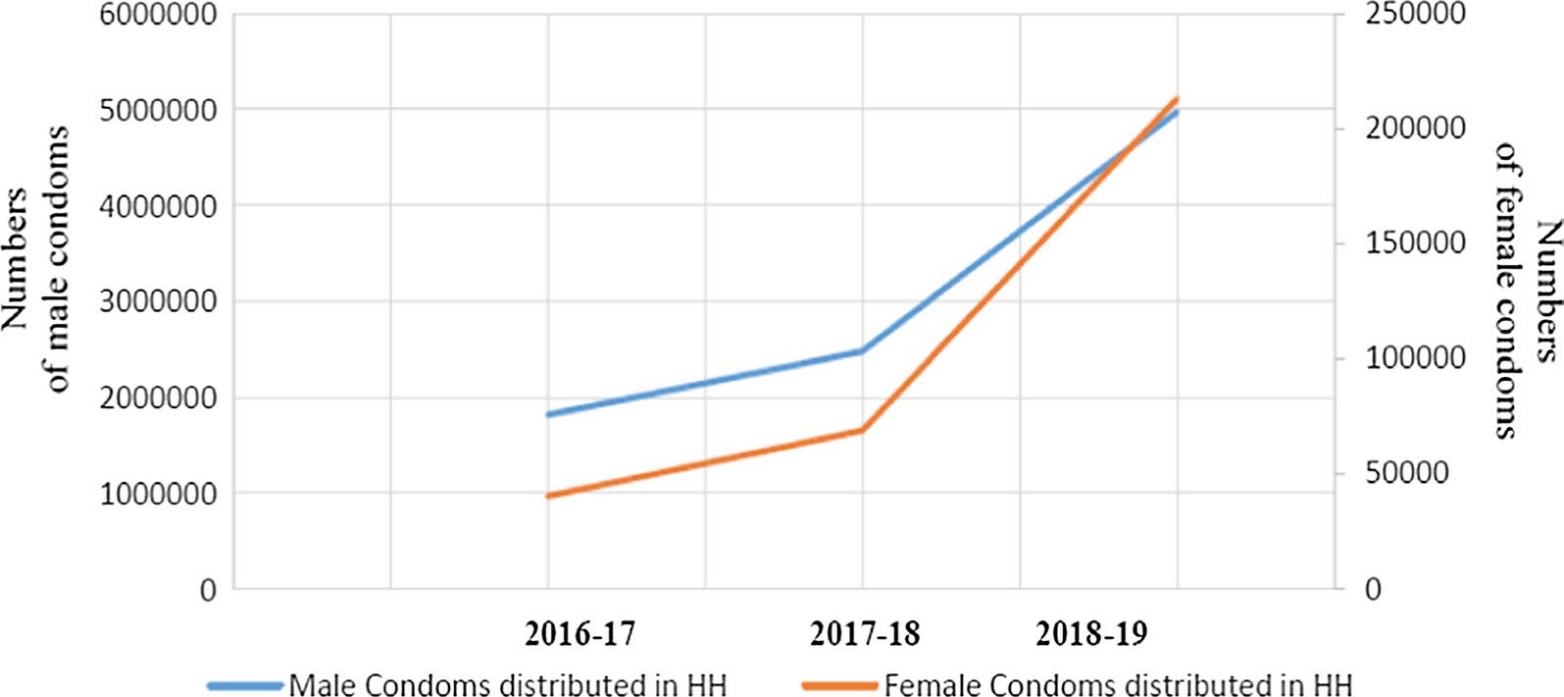
Number of condoms distributed by WBPHCOTs in households

**Fig. 3:**
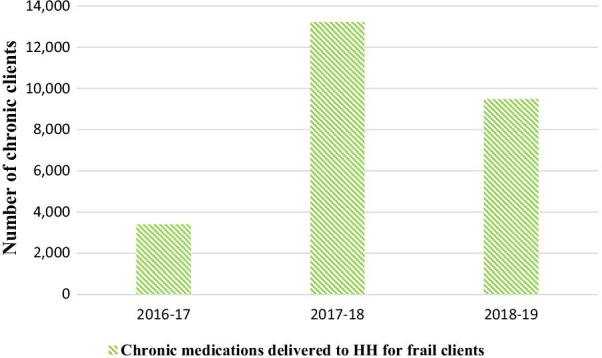
Chronic medication packages delivered to households by WBPHCOTs

**Fig. 4:**
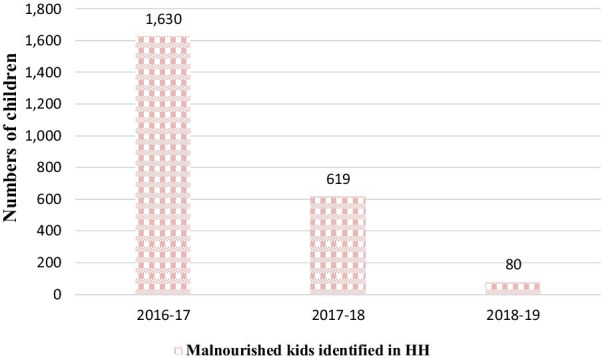
Numbers of malnourished children identified in households by WBPHCOTs

**Fig. 5:**
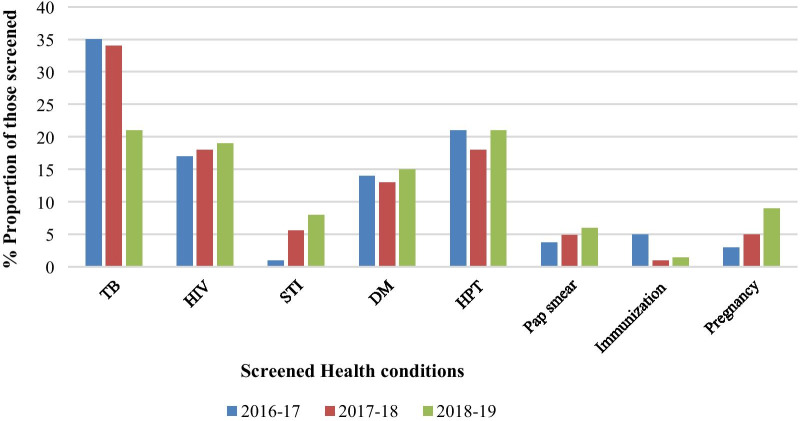
Household screening across health conditions

**Fig. 6:**
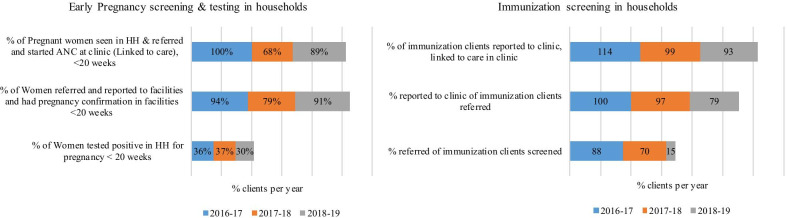
Early pregnancy and child immunization screening in households

**Fig. 7:**
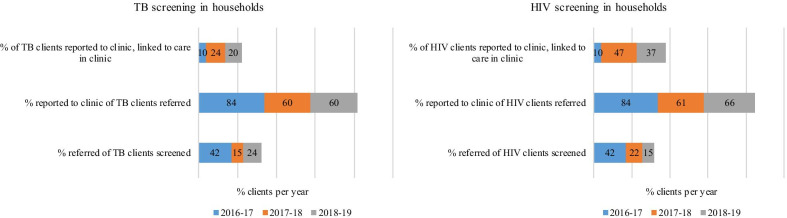
Screening for common infectious diseases (HIV and TB) in households

**Fig. 8:**
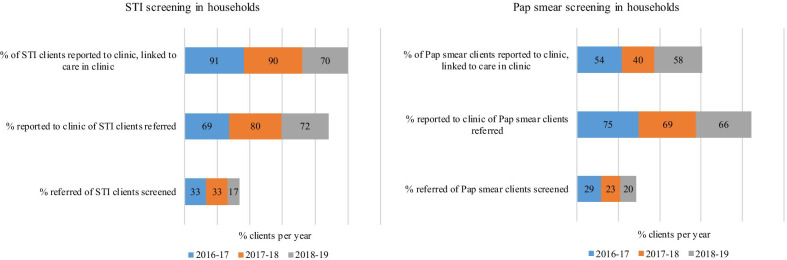
Screening for STI and Pap smear in households

**Fig. 9:**
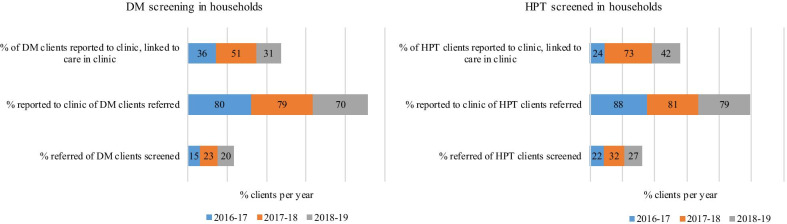
Screening for common non-communicable conditions in households

**Fig. 10:**
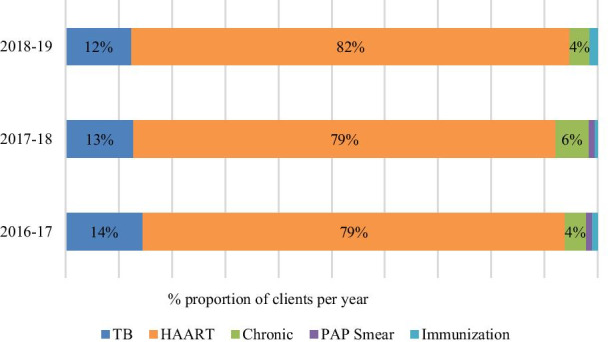
Proportion of clients for tracing by CHWs, per condition

**Fig. 11:**
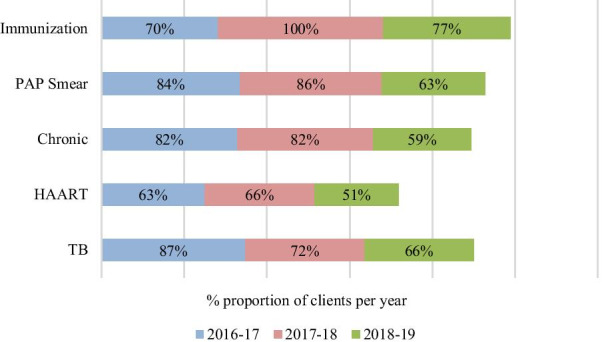
Proportion of clients, per condition, successfully traced in households

**Fig. 12:**
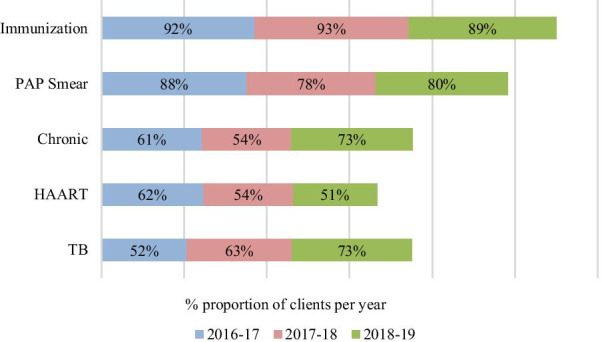
Proportion of clients traced, referred and reported to clinic

**Fig. 13:**
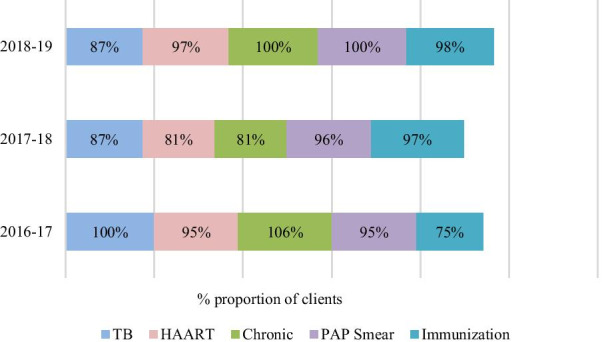
Proportion of traced clients linked back to care, in clinics

**Fig. 14:**
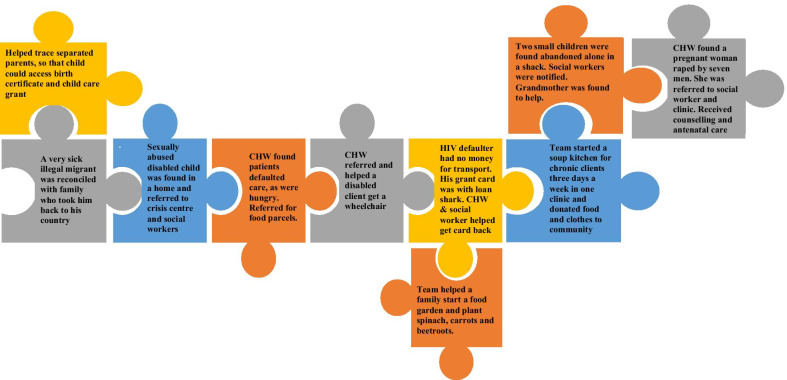
Psychosocial support-touching the lives of those in the communities

- Disease prevention and chronic care:
  As part of a broad range of prevention activities to reduce the risk of HIV and sexually transmitted diseases, condoms were delivered to households. Figure 2 illustrates an increase in condom distribution by more than double for male condoms and a fivefold increase for female condoms over the same period.
  District clinics have many clients with chronic diseases including HIV, TB, hypertension, and diabetes. CHW teams delivered chronic medications to identified households within their catchment areas, especially to clients who were not mobile. The number of clients who received this service grew from 3411 in 2016–2017 to 9494 in 2018–2019 (Fig. 3).
- Household screening activities:
  Screening was conducted in homes using standardized national forms; for infectious diseases (TB, HIV and STIs), non-communicable diseases/NCD (hypertension, diabetes, cervical cancer) and mother and child health problems.
  During household visits, CHWs identified children under five and reviewed their immunization and nutrition status. Children were screened for poor growth (malnutrition) and referred to the nearest clinic. CHWs used a coloured mid-upper arm circumference tape to determine if a child was malnourished or not. Figure 4 illustrates a reduction in the number of malnourished children identified in households covered by WBPHCOTs, from 1630 to 80 malnourished children, over the 3-year period. During this same period numbers of teams were at their most and more households were reached.
  CHWs conducted household screening activities across several health conditions; illustrated in Fig. 5. Although TB and HIV constituted much of the screening, non-communicable diseases contributed significantly too.
  CHWs aim to reduce the burden of mother and child health problems through early household screening for pregnancy (at less than 20 weeks’ gestation), early referral for antenatal care and identification of un-immunized children; Fig. 5 shows that pregnancy screening improved to approximately 10% of all screening activities in 2018–2019. Women of childbearing age were asked if they had a missed period or signs of pregnancy and if so CHWs conducted a rapid urine pregnancy test in the household. Over the period, there was a threefold increase in the overall number of women tested for pregnancy in households, in an attempt to get those at less than 20 weeks gestation. An average of 90% of those testing positive for pregnancy (< 20 weeks gestation) in households reported to clinics, leading to early referrals for antenatal care; Fig. 6.
  CHWs checked the health cards of children to ensure all immunizations were up to date as per national immunization schedules. In CHW supported households, as more children were screened and immunized over the years, there was less need to refer. Figure 6 shows that in 2018–2019 just 15% of children screened had to be referred for incomplete immunization, much lower than previous years. Once mothers and children were referred; 80% reported to the clinics and over 95% were given the required immunization (linked to care). This was due to the emphasis on MCH by CHWs.
  Figure 5 showed that approximately 50% of those screened in households were for HIV and TB; reflecting the emphasis on these priority disease burdens in poor communities. TB screening was based on symptoms of chronic cough, weight loss and night sweats. Over the 3 years, fewer clients required referrals for TB symptoms. HIV screening was based on awareness of HIV status or not. Of those screened and referred for HIV and TB, 60–80% did report to the clinic; improving access to care in vulnerable communities.
  Clients were asked if they had any genital sores or discharges, as part of screening for sexually transmitted diseases (STIs). More than 70% of those who did, reported to clinics and were put on treatment.
  To improve cervical cancer screening, women over 30 years were asked if they had had a pap smear done. This resulted in over 65% of referred women presenting to clinics for a pap smear, Fig. 8. Hypertension (HPT) and diabetes (DM) screening similarly increased over the period under review; contributing to 30 to 40% of health conditions found using clinical symptoms and/or digital BP and glucometer machines. Again, once referred; the proportion of clients who reported to clinics was high, approximately 80%.
  Over the study period, linkage to care in the clinics was good for pregnancy, immunization, STIs and cervical cancer, but less so (< 50%) for those screened for HIV, TB, DM and HPT; Figs. 7 and 9. Linkage to care is when clients are either diagnosed, started on treatment or put back on treatment.
- Defaulter and contact tracing:
  Tracing of contacts or defaulters was another important activity by CHW teams. The clinic head gave teams a list of clients to trace who initially had attended the clinic but failed to come back for either diagnosis and/or start of treatment or were contacts. These clients were often not part of CHW allocated households. Figure 10 illustrates that over 90% of tracing was for defaulting HIV and/or TB treatment.
  Figure 11 illustrates the proportion of clients successfully traced by the CHWs, from the lists provided by the clinics, including clients for whom incorrect contact details were provided. For immunization, cervical cancer and non-communicable disease 70 to 80% of clients were traced on average, despite incorrect addresses and a mobile population, while for TB this was 75% and HIV 60%.
  Many clients traced returned to the clinics for further treatment, Fig. 12. For TB clients and those with chronic conditions (hypertension and diabetes) by 2018–2019 this improved to approximately 70%; while with HIV just 51% of clients returned to the clinics.
  Of those who returned to clinics, over the study period, 90% of contacts and defaulters were linked to care, across the health conditions; Fig. 13.
- Social support activities:

Social support was part of welfare services and not health, but CHWs still played a role in enabling communities to access psychosocial care (Fig. 14).

## Discussion

This study shows the extensive outputs of CHWs and their contributions towards reducing the quadruple burden of disease in the Ekurhuleni district.

Developing countries must consider the role of CHW teams in achieving universal health coverage [21]. Much of the existing research on WBPHCOT programmes in SA explored processes and barriers to implementation [22–25]. Current health service indicators that inform routine monitoring for WBPHCOTs largely document input and process indicators such as numbers of teams, compliance, supervision coverage and households registered [14]. There is little published local evidence on the outputs of WBPHCOT efforts in households in SA.

Ekurhuleni has had high child malnutrition rates compared to other districts in the province, leading to greater emphasis of maternal and child health activities by CHWs. Discussions with OTLs suggest that CHWs did find fewer such children, and routine district indicators showed that between 2011 and 2012, when CHWs were first introduced and 2018–2019, the severe acute malnutrition rate in Ekurhuleni halved, with most of this decline occurring in the latter three years when the district had the most number of teams. Further research is needed to explore team effectiveness.

Most of the 1108 CHWs have supported their households for three years or longer, providing early screening, referrals, access to birth certificates, social grants and food parcels. This role of CHWs aligns with findings documented in several countries [26].

Over the duration of this study period, the numbers of CHWs remained relatively unchanged, however, early antenatal screening still improved in Ekurhuleni; from OTL feedback this was possibly due to gradual improvements in CHW performance. Supervision and support are factors that may have helped, even when there is limited funding and CHWs. Globally, CHWs have demonstrated their impact on improving early antenatal care in poor communities [27]. The increased pregnancy screening, especially in early pregnancy and the identification of unhealthy children in households supported by WBPHCOTs over the study period demonstrates their vital contributions to MCH in Ekurhuleni.

Most CHW screening activities used to be on TB and HIV; these services continued, with fewer clients with TB symptoms being found in CHW households, possibly due to the early TB screening, referrals and treatment. Since 2011–2012 a wider range of health conditions are part of the WBPHCOT household screening activities in Ekurhuleni as compared to the past. Infectious diseases and NCDs are commonly seen in both urban [28] and rural areas [29] related to the growing aging population and the quadruple burden of disease in the country. Given these demographic changes, the role of CHWs in early household screening, referrals and treatment support of hypertension, diabetes and cervical cancer becomes crucial. They can increase early chronic disease diagnosis, improve chronic disease control and reduce complications over time.

Early and appropriate referrals to primary health care services is an important part of access to care. CHWs can make appropriate neonatal or pregnancy referrals [30, 31]. Through better education about immunizations and identification of defaulters, over time there were less children to refer, improving child immunization in CHW supported households [32].

In addition to screening, clients that require care must be referred to PHC clinics. In our study, those with NCDs and requiring immunization had the highest reporting rates to clinics. CHWs are better able to explain to clients reasons for referral, promoting the client to report to the next level of care [33]. CHWs influence the health seeking behaviour of mothers and child caregivers, resulting in good reporting to clinics, and contribute to overall district performance on immunization [33]. Evidence of this in Ekurhuleni is that 60–80% of clients referred by CHWs reported to the clinic, facilitating access to care.

Once CHW referred clients report to clinics, they are seen by a clinician. As clients reported to clinics, the health service responded through further investigations, or diagnosis and/or management of conditions. OTLs engaged clinicians on their clients’ behalf, checked the daily number of CHW referrals with those who reported to the clinic and accessed care. Clients referred for pregnancy and immunization had good linkage to care in Ekurhuleni over the years, partly due to fewer numbers and increased clinic staff acceptance of the role CHWs played in early screening and referrals. There was reasonable linkage to care for those with STIs and possible cervical cancer. OTLs played a vital role in enabling access to care. However, not as many NCD, TB and HIV clients were linked to care, possibly due to larger numbers, inferring that the capacity of the health service to respond to CHW referrals was an ongoing challenge [34].

Physical tracing of defaulter clients in an urban setting in South Africa, is not without its challenges, especially for HIV and TB defaulters. With a large migrant population in urban settings, there are challenges in getting the correct contact details of clients. This problem is reiterated in studies conducted in Kenya [35] and other parts of South Africa [36]. In Ekurhuleni some patients deliberately provide false contact details, while others seemingly move around frequently for jobs and other reasons. This is particularly so for HIV defaulters, as shown in Fig. 11, where only 50–60% of defaulting clients were successfully traced. South Africa has one of the largest anti-retroviral programmes in the world and is struggling to achieve the 90-90-90 targets for initiation of anti-retroviral treatment and retention in care [37]. Although CHWs contributed in finding defaulting HIV clients in Ekurhuleni, to improve this further, there is a need to record accurate patient information when accessing care the first time, updating information regularly and/or getting details of a second contact known to the client [35]. Another factor is stigma around an HIV diagnosis with many patients not disclosing their status to their partners [38]. Many defaulters were also in households not allocated to CHWs and of those they successfully traced, a good proportion did report to clinics. This implied good CHW communication skills [35]. Greater household support and contact with CHW teams has the potential to reduce stigma and defaulters in the long-term.

A large proportion of defaulters who reported to clinics were put back on treatment/care; this positive finding was possibly due to a combination of clinics initiating tracing, awareness by clinic staff of returning patients and less clinical work-up needed. CHWs consistent and improved performance may have also contributed to the improved return to care.

CHWs have the opportunity to provide wider support on the psycho-social determinants of health in these communities, though this was not easily quantified. Where households could be directed to get a birth certificate for a child or an identity document for an elderly person, it meant access to financial support through various welfare grants in South Africa. Accessing these grants would mean access to food, money for medicines, schooling and other essentials [39]. Even though South Africa emphasized a primary health care approach in health policies from 1994; PHC delivery was more curative and clinician driven, with less emphasis on psycho-social services [40]. In discussions with CHW teams they emphasized what they believed is their critical contribution to addressing psycho-social challenges, however no data is collected to support this [41]. Provision of social support reinforces the credibility of CHWs in households leading to improved household relationships [42]. The mechanisms by which CHWs influence heath behaviour change over time, possibly through this social support, are part of the puzzle of understanding their role, Fig. 14. Future research should explore this aspect further.

Over the years, Ekurhuleni clinics have realized the value of CHW teams as the foot soldiers of the health service within the community. This perception translates to believing that they are the solution to most community related challenges affecting the health system. While the results of this study did not explore nor indicate this, evidence shows that overloading CHWs with too many activities can make them inefficient in their daily tasks [43]. Current workloads with simple tasks and messages across health conditions are reasonable for optimized performance.

There was some reduction in performance of team activities during 2018–2019. This is likely to have been caused by two issues. The district was a pilot site for a national standardized training program. Secondly, CHWs were agitating to be made permanent staff. Both these affected household visits in that year. In April 2020, the CHWs in Ekurhuleni were made permanent with salaries, benefits and job security; so performance is expected to improve in subsequent years.

Limitations of this study include that we did not explore quality or retention in care. And although we did not also compare with other districts, South Africa publishes an annual district health barometer each year, which showed that Ekurhuleni had improved in many areas of maternal, child health, TB and HIV [44].

## Conclusion

The extent of CHW activities is not captured in official routine reports nor documented in local or global studies. This study has illustrated the extensive contributions to care made through a large-scale CHW program on an estimated one million population in 280,000 households in an urban metropolitan district in South Africa. The findings also show that CHWs have managed to easily move from a HIV/TB focus to providing a comprehensive range of services across health and social conditions. This supports evidence that CHW programmes can enable greater access to care in vulnerable communities through their own efforts and through referrals to clinics; thus, contributing towards universal health coverage in SA.

## Recommendations

CHW outputs across health conditions such as maternal and child care (pregnancy screening, antenatal care support, immunization screening, monitoring child nutrition), chronic diseases (hypertension and diabetes education, screening and support) and HIV/TB and other infectious diseases (screening, support, education) have been demonstrated and should be part of the standardized scope of CHW work nationally.

The clear evidence of the extensive contribution by CHWs in Ekurhuleni illustrates the need to expand these teams, as per national norms. We therefore recommend for a significant increase in the number of CHW teams to cover more areas in the district and country.

With their high value in terms of benefits and low cost, CHW teams are a good investment of public funds. If additional funding cannot be secured, staggered implementation and re-prioritization of other services and resources to fund additional teams should be considered.

## Supplementary Information

**Additional file 1: Appendix S1.** List of Abbreviations.

**Additional file 2: Appendix S2.** Map of South African health districts and Ekurhuleni specifically.

**Additional file 3: Appendix S3.** Template of monthly team reports.

## Availability of data

On reasonable request after completion of all related studies.
