# Supplementary material for: An analysis of the services provided by community health workers within an urban district in South Africa: a key contribution towards universal access to care
Source: Hum Resour Health. 2021 Feb 18;19:22. doi: 10.1186/s12960-021-00565-4 (PMC7889710; doi:10.1186/s12960-021-00565-4)
Supplement: Supplementary file 1 — Additional file 1: Appendix S1. List of Abbreviations. [file 12960_2021_565_MOESM1_ESM.docx]

| AIDS | Acquired Immuno-deficiency syndrome |
| --- | --- |
| ANC | Antenatal care |
| CHW | Community Health Worker |
| DM | Diabetes Mellitus |
| EN | Enrolled nurse |
| HAST | HIV, AIDS, STI, TB |
| HH | Households |
| HIV | Human Immunodeficiency Virus |
| HPT | Hypertension |
| MCH | Mother and Child Health |
| NCD | Non-communicable diseases |
| NGO | Non-governmental organisation |
| NHI | National Health Insurance |
| OPD | Outpatients Department |
| OTL | Outreach Team Leader |
| PHC | Primary Health Care |
| PN | Professional nurse |
| SA | South Africa |
| STI | Sexually transmitted Infections |
| TB | Tuberculosis |
| WBPHCOT | Ward based PHC Outreach team |
| WHO | World Health Organisation |
