# Supplementary material for: An analysis of the services provided by community health workers within an urban district in South Africa: a key contribution towards universal access to care
Source: Hum Resour Health. 2021 Feb 18;19:22. doi: 10.1186/s12960-021-00565-4 (PMC7889710; doi:10.1186/s12960-021-00565-4)
Supplement: Supplementary file 3 — Additional file 3: Appendix S3. Template of monthly team reports. [file 12960_2021_565_MOESM3_ESM.docx]

**District:**

**Month:**

**Number of Teams:**

**No of OTLs:**

**Number of CHWs:**

**1. Key Priorities**

| **Number of male condoms distributed:** |  |
| --- | --- |
| **Number of female condoms distributed:** |  |
| ***Number of clients with chronic medications for home delivery:** |  |
| ***Number of malnourished children identified:** |  |

** Submit separate list with names and addresses, and condition*

**2. In-service training of CHWs**

| **Date** | **Topic** |
| --- | --- |
|  |  |
|  |  |
|  |  |
|  |  |
|  |  |
|  |  |

**3. Courses attended by OTLs**

| **Date** | **Course attended** |
| --- | --- |
|  |  |
|  |  |

**4. Campaigns Participated**

| **Date** | **Campaign** | **Activities** | **Referral to clinic** | **Reasons for referral** |
| --- | --- | --- | --- | --- |
|  |  |  |  |  |
|  |  |  |  |  |

**5. Support Groups Facilitated:**

| **Date** | **Group Name** | **Topic** | **No. of Clients** | **Facilitator** |
| --- | --- | --- | --- | --- |
|  |  |  |  |  |

**6. Screening done**

| **Activities** | **No. screened** | **No. Referred** | **No. Reported at the Facility** | **Link to care/Outcome** |
| --- | --- | --- | --- | --- |
| **TB** |  |  |  |  |
| **HIV** |  |  |  |  |
| **STI** |  |  |  |  |
| **Pregnancy** |  |  |  |  |
| **Family Planning** |  |  |  |  |
| **Immunisation** |  |  |  |  |
| **Vit A & Deworming** |  |  |  |  |
| **Malnutrition** |  |  |  |  |
| **Diabetes** |  |  |  |  |
| **Hypertension** |  |  |  |  |
| **Pap Smear** |  |  |  |  |
| **MMC** |  |  |  |  |
| **Social support services** |  |  |  |  |
| **Cataract** |  |  |  |  |

**7. Defaulters Traced/Lost to follow up**

| **No. of Defaulters to trace** | **No. successfully traced** | **Reported to clinic** | **Total Linked to care** |
| --- | --- | --- | --- |
| **TB =** |  |  |  |
| **ART=** |  |  |  |
| **Pap smear=** |  |  |  |
| **Mental Health=** |  |  |  |
| **Chronic=** |  |  |  |
| **Bloods =** |  |  |  |

**9. Pregnant Women:**

|  | **< 20 weeks** | **>20 weeks** |
| --- | --- | --- |
| **No. Tested for Pregnancy** |  |  |
| **No. Tested positive for Pregnancy** |  |  |
| **Confirmed test at the Facility** |  |  |
| **No. Started ANC** |  |  |

**ACHIEVEMENTS**

**CHALLENGES**

**COMPILED BY:**

**Date:**
