# Supplementary material for: An analysis of the services provided by community health workers within an urban district in South Africa: a key contribution towards universal access to care
Source: Hum Resour Health. 2021 Feb 18;19:22. doi: 10.1186/s12960-021-00565-4 (PMC7889710; doi:10.1186/s12960-021-00565-4)
Supplement: Supplementary file 3 [file 12960_2021_565_MOESM2_ESM.docx]

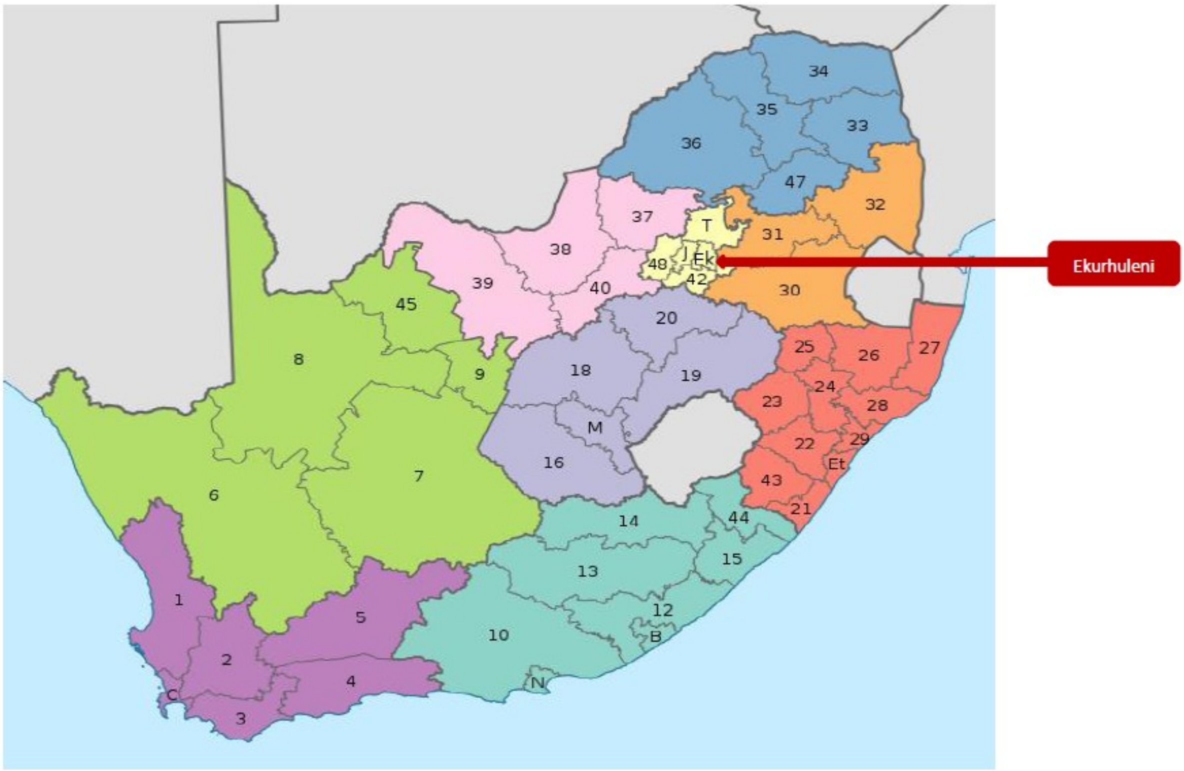
Ekurhuleni
34
35
33
36
47
32
37
T
31
38
J
Ek
48
42
39
40
30
45
20
8
25
26
27
18
9
19
24
23
28
M
22
29
Et
16
7
43
6
21
44
14
15
13
1
5
12
10
B
2
4
N
C
3
